# A puzzle related to superlative modification in definite relative clauses in Jordanian Arabic

**DOI:** 10.1016/j.heliyon.2022.e10115

**Published:** 2022-08-14

**Authors:** Abdel-rahman Abu Helal

**Affiliations:** College of Arts, Department of English Language and Literature, Al-Zaytoonah University of Jordan, P.O.Box 130, Amman 11733, Jordan

**Keywords:** Absolute/Relative superlatives, Definite relative clauses, Late merger

## Abstract

This paper addresses a puzzle involving two modes of superlative modification in definite relatives in Jordanian Arabic^1^: the so-called (A)ttributive and (G)enitive superlatives.^2^ We show that under the low interpretation of the definite relative clause, both modes of superlative modification exhibit an asymmetrical behavior: while the A-superlative is ambiguous between the absolute and relative readings, the relative reading is blocked in G-superlatives. To resolve the puzzle, we propose a compositional analysis based on the late merger of the definite relative which generates the absolute/relative ambiguity in A-superlatives and at the same time it blocks the relative reading in G-superlatives.

## 1 Introduction

### 1.1 Two forms of superlative construction in Jordanian Arabic^3^

The standard degree-based theory has two main working assumptions about gradable adjectives: first, gradable adjectives (e.g., tall) denote relations of individuals and degrees (Seuren, 1973; Cresswell 1976; von Stechow 1984; Heim, 1985, 2000) as represented in (1).

**Table:** 

(1)	⟦ tall ⟧ = λd.λx. HIGHT (x) ≥ d

Second, this relation is downward monotonic with the following property (Romero 2013):

**Table:** 

(2)	∀x∀d∀d'. [ HIGHT (d) (x) = 1 ∧ d' < d → HIGHT(d’) (x) = 1]

Syntactically, the degree argument of gradable adjective is represented as a DegP that is projected as sister to the gradable adjective. This degree argument composes in two ways: first, it can be directly saturated by degree-denoting measure phrases of typed which combines with the gradable adjectives via function application (Heim 1985). Alternatively, it can be quantified over by a degree operator. One of these degree operators is the superlative morpheme –est.^4^ A possible lexical entry for the superlative operator –est is a two-place predicate as defined in (3) (Heim 2000).^5^

**Table:** 

(3)	⟦ -est ⟧ = λG_<dt,t>_ λP_<dt>_. ∃d [ P(d) ∧ ∀Q ∈ G [ Q ≠ P→ ¬ (Q(D))]

The superlative operator in (3) compares the set of degrees originated in the matrix clause with the sets of degrees denoted by the contextually-determined comparison class C and it asserts that the relevant individual has the scalar relation to a degree d that no other element in the comparison class has (Heim 1999). For example, the superlative in (4) has the absolute reading in which the speaker compares articles in terms of their length, picks the longest one among a contextually-determined set of articles and asserts that Ali read that article (Heim 1999).

**Table:** 

(4)	*ʕali*	*ɡara*	*l-maqalah*	*l-ʔtˤwal*
	Ali	read	the-paper	the-longest
	'Ali read the longest article.'			

The superlative construction in (4) has another reading; the so-called relative reading (Ross 1964; von Stechow 1984; Szabolcsi, 1986; Heim 1985, 1999; Rullmann 1995; Romero 2011). On the relative reading, the speaker compares Ali to other article-readers in terms of their reading achievements and asserts that Ali read a longer article than anyone among contextually relevant readers read.

Under the head-external view (Heim 1999, Szabolcsi, 1986),^6^ the absolute/relative ambiguity is derived through the construction of two LF structures. That is, the LF position of the superlative operator [-est C] determines the domain of possible comparison classes of C.^7^ On the absolute reading, the [-est C] moves minimally within the DP *the longest article* and it creates a degree abstract over the degree d in its original position. The LF that delivers the truth conditions of the absolute reading has the following representation:

**Table:** 

(5)	**Absolute Reading**: Ali read the longest article.	
	a.	–est QRs and the N′-internal PRO that moves and deletes leaving a trace t_2,F_ of type e that is focus-marked]
		LF: Ali read THE 2 [ [ -est C] [ 1 [ t_2,F_ t_1_-long article ]] ∼ C
	b.	Ali read the unique x: ∃d [ article (x) & long (x,d) & ∀Q ∈ C [ Q ≠ (λd’. x is a d’-long article) → ¬ Q(d)]

On the relative reading, the [-est C] moves out of the DP *the longest article* (i.e., minimally within the VP *read the longest article* and again it creates a degree abstract over the degree d in its original position as represented in (6).

**Table:** 

(6)	**Relative Reading**: Ali read the longest article.	
	a.	LF: [ [ -est C] [ 1 [ BILL_*F*_ read a t_1_-long article ]] ∼ C
	b.	∃d ∃x [ article(x) & long (x,d) & read (x,d) ]& ∀Q ∈ C [ Q ≠ (λd’. Ali read s a d’-long article) → ¬ Q(d)]

In Jordanian Arabic, the superlative adjective is formed by replacing the three consonantal root cluster of the positive adjective ‘C_1_C_2_C_3_'with the template sequence ‘ʔC_1_C_2_ aC_3_’ (Hallman, 2016; Grano & davis 2018). For example, the positive form *tˤawiil* ‘tall’ with its root cluster *tˤwl* has the superlative form *ʔtˤwal* ‘taller’.^8^

Superlative adjectives (e.g., *ʔtˤwal* ‘taller’) may follow or precede the noun it modifies as in (7). The two forms of superlative structure vary with respect to the morphosyntax of definiteness and Case: when the superlative adjective post-modifies the noun, both the noun and the post-modifying superlative adjective have the overt definite article *l* as in (7.a). By contrast, the pre-modifying superlative adjective never incorporates definite morphology as shown in (7.b).

**Table:** 

(7)	a.	*∗(l-)maqalah*	*∗(l-)ʔtˤwal*	*yalli*	*Einstein*	*katab-ha*
		∗(the-)article	∗(the-) longest	Rel	Einstein	wrote-it
		Literal: ' The article the longest that Einstein wrote '				
		≈: ' The longest article that Einstein wrote '				
	b.	*(∗l-)ʔtˤwal*	*(∗l-)maqalah*	*yalli*	*Einstein*	*katab-ha*
		(∗the-)longest	(∗the-) article	Rel	Einstein	wrote-it
		Literal: ' Longest article that Einstein wrote '				
		≈: ' The longest article that Einstein wrote '				

When it comes to Case, the post-modifying superlative adjective agrees with the NP in Case feature. As in (8), the post-modifying superlative adjective inherits the Case feature of the NP head.

**Table:** 

(8)	a.	*l-tˤaalib*	*l-ʔtˤwal*	*rasab*
		the-student	the-tallest	failed
		Literal: ' The student the tallest failed. '		
		≈: ' The tallest student failed. '		
	b.	*nadʒdʒaħ*	*l- tˤaalib*	*l-ʔtˤwal*
		I passed	the-student	the-tallest
		Literal: I passed the student the tallest.		
		≈: ' I passed the tallest student. '		

On the other hand, the head NP that is pre-modified by the superlative adjective bears a default genitive Case mark as in (9) (See Abu Helal, 2022 and Elghamry (2003) for evidence from Levantine and Standard Arabic, respectively).^9^

**Table:** 

(9)	a.	*ʔtˤwal*	*tˤaalib*	*rasab*
		tallest	student	failed
		Literal: ‘tallest student failed.’		
		≈: ‘The tallest student failed.’		
	b.	*nadʒdʒaħt*	*ʔtˤwal*	*tˤaalib*
		I passed	tallest	student
		Literal: ' I passed tallest student. '		
		≈: ' I passed the tallest student. '		

On a minimalist view to Case (Chomsky, 1995, 1998), structural Case assignment is achieved by a Goal-Probe Agree operation based on c-command relation. Accordingly, the Case- marked expression that enters the derivation with an unvalued Case feature serves as Goal for a c-commanding functional head which serves as Probe that values the Case feature of that expression by virtue of agreement (i.e., in phi-features such as person, number, and gender).^10^

Given these observations, it should be the case that the two superlative structures in (9.a) and (9.b) have different Case assignment domains: while the nominative and accusative Case is valued at the sentential level via an Agree operation with the c-commanding tensed T^0^ and transitive v^0^, respectively, the genitive Case is valued within the DP structure via an Agree operation with the c-commanding D^0^ as schematized in (10) and (10)’ (Siloni 1997 and the references therein).

**Table:** 

For the ease of exposition, we will refer to the first form of superlative structure in (9.a) as A(ttributive) superlative and the second form in (9.b) as G(enitive) superlative.

### 1.2 The puzzle

In Jordanian Arabic, when an adjectival modifier such as the superlative morpheme composes with the *yalli* relative clause that dominates a propositional attitudinal verb, the so-called high/low ambiguity of the relative clause arises.^11^ Consider, for example, the superlative structure in (11) which is ambiguous between the high and low readings of the *yalli* relative clause as the result of the A- and G- superlative modification (See Abu Helal 2022).

**Table:** 

(11)	*ʕali*	*ɡara*	*l-maqalah*	*l-ʔtˤwal/*	*ʔtˤwal*	*maqalah*
	Ali	read	the-article	the-longest/	longest	article
	yalli	Aħmad	ħaka	ʔinnu	Einstein	katab-ha
	Rel	Ahmad	said	that	Einstein	wrote-it
	Literal: 'Ali read the article the longest/longest article that Ahmad said that Einstein wrote. '					
	≈: 'Ali read the longest article that Ahmad said that Einstein wrote. '					
	**High Reading**					
	Einstein's photoelectric effect article is actually 17 pages and his Special Relativity one is actually 31 pages. Therefore, the relative clause structure in (11) refers to the Special Relativity article, which is the actual longest paper about which Ahmad said that Einstein wrote.					
	The superlative in (11) refers to the longest paper about which Ahmad said that Einstein had written it					
	**Low Reading**					
	Ahmad said that Einstein's photoelectric article is 20 pages long and his Brownian motion is 12 pages. Therefore, the longest paper is Einstein's paper on photons.					
	The superlative in (11) refers to the x such that Ahmad said that x was the longest paper written by Einstein					

However, the two forms of superlative exhibit a set of interpretive asymmetries when modified the *yalli* relative dominating attitudinal predicate. First, the A-and G-superlatives are ambiguous between the absolute and relative interpretations under the high reading as follows.

**Table:** 

(12)	**high reading:**		
	(absolute)	✓ A-superlative	✓ G-superlative
	It is true iff Ali read an article that is long to a degree to which no other actual article about which Ahmad said that Einstein wrote is long.		
	(relative)	✓ A-superlative	✓ G-superlative
	It is true iff Ali read an article x that is long to a degree d such that no other individual read an actual article to degree d about which Ahmad said that Einstein wrote.		

Under the low reading, the A-superlative is also ambiguous between the absolute/relative interpretations. But when it comes to the G-superlative the relative reading is blocked as described in (13).

**Table:** 

(13)	**Low reading**		
	(absolute)	✓ A-superlative	✓ G-superlative
	It is true iff Ali read an article that is long to a degree to which no other article in Ahmad's saying -worlds is long.		
	**(relative) ✓ A-superlative**	✗ **G-superlative**	
	It is true iff Ali read an article that is long to a degree d such that no other individual read an article in Ahmad's saying –worlds to degree d.		

The facts in (12) and (13) present a puzzle: why is the relative low reading of G-superlatives blocked and such a reading is available in A-superlatives?! This paper aims at analyzing the two forms of superlative modification under the low reading of the *yalli* relative. More specifically, it proposes a unified analysis that explains the semantic puzzle represented by the facts in (12) and (13).

The paper is structured as follows. Section 2 reviews the representative literature on adjectively modified relative clauses. It shows that a raising syntax is required for the presence of the low/high ambiguity (Bhatt 2002; Hulsey and Sauerland 2006). Section 3 motivates the working assumption of our proposal. It argues for the claim that the *(ya)illi* relative clause modified by the superlative is a matching structure with an amount interpretation that serves as the complement comparison class of the QRing superlative operator by later merger. Section 4 presents the desired unified compositional analysis that resolves the puzzle. The last section concludes the paper.

## 2 A tale of two ambiguities

### 2.1 The head external vs. head raising analyses

Recent grammatical investigations into relative clauses have examined their properties with respect to two cases of structural ambiguity. The first case is a syntactic ambiguity in which relative clauses such as the *yalli* relative in (14) may be analyzed relative to two main lines of analysis: the head external and head NP raising structures.

**Table:** 

(14)	*l-maqalah*	*yalli*	*Einstein*	*katab-ha*
	the-article	Rel	Einstein	wrote-it
	'The paper that Einstein wrote'			

On the head external analysis, the head NP is base-generated outside the relative clause and an internal relative clause operator undergoes A-bar movement as in (15). The operator's movement creates a predicate of individuals that composes with the NP via predicate modification. In this way, the external NP head has no representation within the relative clause (Chomsky, 1977).

**Table:** 

A variant of head external analysis is the matching structure in (16). On the matching analysis, the head NP is base generated in the left periphery of relative clause and an internal representation of the NP gets interpreted within the relative clause through an operation of ellipsis-based movement deletion under identity. Therefore, the head NP and its internal representation are not connected via movement chain so that the external NP doesn't exhibit reconstruction effects. It is still represented within the relative clause by a copy that is left by movement deletion. Such a copy is identical or partially similar to the external NP head (Sauerland, 1998 and subsequent work).

**Table:** 

The head NP raising analysis, on the other hand, involves a raising syntax where the relativized NP undergoes A-bar movement from a relative-clause internal position into its surface landing site in the left periphery of the relative clause in (17). Given A-bar movement, the head NP reconstructs and gets interpreted in its base-generation position within the relative clause (Schachter 1973; Verngaud 1974; Kayne 1994; Afarli 1994; Bhatt 2002, among many others).

**Table:** 

### 2.2 The high/low ambiguity

The other case of ambiguity arises when an adjectival operator (e.g., the superlative –est) modifies relative clauses that dominate a propositional attitudinal verb as exemplified in (18) (Bhatt 2002; Hulsey and Sauerland 2006; Heycock 2005).

**Table:** 

(18)	the longest book that John said that Tolstoy had written	
	**‘High’ reading:**	
	x is the longest book out of the books about which John said that Tolstoy had written them.	
	**‘Low’ reading:**	
	What John said can be paraphrased as ‘x is the longest book that Tolstoy wrote.	
		(Bhatt 2002: 57, Heycock 2005: 360)

As argued in Bhatt (2002) and Hulsey and Sauerland (2006), the low readings of the relative clause in (18) provide an argument for the assumption that the head NP has an internal origin within the relative clause. Bhatt (2002) proposed that the low readings of (18) involve the reconstruction of the head NP within the relative clause based on the head raising analysis. For example, the external NP with its adjectival modifier –est in (18.b) is claimed to perform successive cyclic movement which forms a chain of copies that can be interpreted in different left-peripheral positions. On the low reading, the –est operator undergoes QRing within the embedded clause under the scope of the propositional attitude verb as represented in (19).^12^

**Table:** 

In (19), the –est operator compares the predicate of degrees [λd. Tolstoy wrote the d-long book x] with the predicates of degrees denoted by a contextually-determined comparison class C and it asserts that the relevant book has a length to a degree d that no other element in the comparison class has as represented in (20).^13^

**Table:** 

(20)	the λx [that John said that –est λd [ Tolstoy wrote [ the d-long book x]]]	
		(Bhatt 2002: 65)

The matching analysis of (20) is represented by the following tree (21).

**Table:** 

This tree has the following output:

**Table:** 

(22)	the [ longest book] λx [John said that –est λd [Tolstoy had written [ the d-long book x ]]]	
		(Bhatt 2002: 67)

As shown in (Bhatt 2002), the predicate of individuals ‘λx [that John said that –est λd [Tolstoy wrote [ the d-long book x]]]’ directly saturates the definite description operator in the raising structure (20). The same predicate of individuals composes with the externally base-generated NP head ‘longest book’ in the matching structure in (22) and the resulting output saturates the definite description operator.

Bhatt (2002) argued that it is the raising structure in (20) that may generate the low reading of the adjectival modifier, rather than the matching structure in (22). In the situation where John said that *War & Peace* was the longest book written by Tolstoy, we want the predicate ‘[longest book] λx [John said that –est λd [Tolstoy had written [ the d-long book x ]]]’ to denote the singleton set consisting of *War & Peace.* Since this set has a unique element in its denotation, it can compose felicitously with the definite description operator.

The composition, however, may wrongly produce unattested readings. Consider the situation where John made several claims about which book of Tolstoy was the longest from time to time (e.g., Last week, John said that *the Cossacks* was the longest book written by Tolstoy and yesterday he said that *War and Peace* was the longest). Since the predicate of individuals, ‘[λx [that John said that –est λd [Tolstoy wrote [the d-long book x]]]’, which can be taken to refer to the set of books that John claimed to be the longest, may compose directly with the NP ‘*longest book*’ to yield a uniquely singleton set that can saturate the definite description, it can be judged felicitous in this situation, meaning that the entire DP doesn't actually has the denotation that the x such as John said that the longest book written by Tolstoy was x, which expresses the low reading.

The raising structure, on the other hand, correctly rules out this unattested reading. On the assumption that the predicate of individuals ‘[λx [that John said that –est λd [ Tolstoy wrote [ the d-long book x]]]’ denotes a set of books that were claimed by John to be Tolstoy's longest from time to time, such a predicate would induce a presupposition failure when composing with the definite description: it violates the uniqueness presupposition. As such, Bhatt (2002) concluded that the raising structure is required for generating the low reading.

In a similar vein, Hulsey and Sauerland (2006) analyzed the high/low ambiguity in terms of the binding of world argument. In (23), the high reading refers to the longest actual book amongst an actual set of books which John believes that Tolstoy wrote. The low reading refers to the book in John's belief worlds such that John believes that it is Tolstoy's longest.

**Table:** 

(23)	The longest book John believes Tolstoy wrote

On Hulsey & Sauerland's analysis, the matching structure as represented in (24) may not derive the low reading.

**Table:** 

(24)	λw …. the –est(C) λd [ long(d) (w) and book(w), λx John believes (w) λw’ Tolstoy wrote the_x_ long (d) (w’) book(w’)]

This is the case because the definite description that corresponds to the trace of movement deletion ‘the_x_ long (d) (w’) book(w’)’ induces a presupposition failure: it presupposes the existence of a unique pair (x,d) such that the book x is actually long to the degree d. But since the structure cannot be false for any (x,b) if d is longer than x's actual length, the definite description results into a presupposition failure and hence it renders the second argument of –est operator (i.e., the predicate of degrees ‘λd [ long(d) (w) and book(w) …….. the_x_ long (d) (w’) book(w’)]) undefined for the sake of comparison.^14^

Hulsey and Sauerland (2006) claimed that a raising analysis is in order. They propose a raising syntax that makes use of the following steps as represented in (25): first, the head NP undergoes successive cyclic movement. Second, the adjective *longest* is adjoined in the left periphery of the embedded clause ‘[Tolstoy wrote the_x_ book(w’)]’. Third, the world lambda operator λw’ of the intensional verb ‘believe’ applies via intensional function application (Heim and Kratzer 1998). Finally, the –est operator undergoes movement into a higher scope position under the scope of the world lambda operator of the intensional verb. In this way, the raising analysis in (25) generates the low reading: there is a unique book x such that John believes it is Tolstoy's longest.

**Table:** 

The conclusion that can be drawn from this section is that the low reading of relative clause requires a reconstruction-based analysis, meaning that a semantic system based on the head external or matching analyses may not generate the low reading of adjectival modifiers of relative clauses.^15^

## 3 The proposal

### 3.1 The yalli relative clause as head external structure

Since the modified relative clause triggers the high/low ambiguity, it should be the case that the *yalli* relative clause is expected to have a head-NP raising analysis. Ouhalla (2004) and Aoun et al. (2010) extended a head-raising analysis to the *yalli* relative clause along the lines of (26).^16^ They postulate a raising syntax where the head NP raises into the Specifier of the relative head D^0^ on the basis of definiteness agreement.^17^

**Table:** 

As argued in Abu Helal (in press), the *(ya)illi* relative clause has in addition to the head NP raising analysis (26) a head external structure (e.g., matching). Abu Helal (in press) argued that the *yalli* relative clause is ungrammatical in contexts where the NP head raising is required.^18^

One piece of evidence comes from the interpretation of idiomatic expressions such as (27).

**Table:** 

(27)	a.	*ʕali*	*ʔkal*	*hawa.*	*kul-na*	*xufna*	*ʕaali-h*
		Ali	ate	air.	All-us	concerned	on-him
		'Ali ran into troubles. All of us concerned about him. '					
		'Literally: Ali ate the air. We concerned about him. '					
	b.	*∗l-hawa*	*xawwf-na*	*ʕala*	*ʕali*		
		The-air	concerned-us	on	Ali		
		'The trouble Ali had made us concerned about him. '					
		Literally: 'The air made us concerned about him. '					
	c.	*∗ l-hawa*	*yalli*	*ʕali*	*ʔkal-u*	*xawwf-na*	*ʕaali-h*
		The-air	Rel	Ali	ate	concerned-us	on-him
		'The trouble that Ali had made us concerned about him. '					
		‘ Literally: The air that Ali ate made us concerned about him.' (Abu Helal 2021a: p. 8)					

The ungrammaticality of (27.c) is unexpected if the *yalli* relative clause is taken to be a raising structure: a head NP raising structure would wrongly rule in (27.c) given that the locality condition of constituency in the idiomatic expressions would be satisfied under reconstruction.

Further, the following binding-theoretic facts present even stronger evidence that argues against head NP raising in the *yalli* relative clause in PA.

**Table:** 

(28)	a.	*ʕali*_*i*_	*ʕallaɡ*	*sˤurah*	*la*	*ħalu*_*i*_	*fi*	*l-maktab*		
		Ali_*i*_	hanged	picture	of	himself_*i*_	in	the-office		
		' Ali put a picture of himself in the office.'								
	b.	∗ʃuft	sˤurah	la	ħalu_i_	yalli	ʕali _i_	ʕallaɡ -ha	fi	l-maktab
		I saw	picture	of	himself_*i*_	Rel	Ali_*i*_	hanged-it	in	the-office
		' I saw a picture of himself that Ali hanged in the office.'

**Table:** 

(29)	a.	*∗ʔaya*	*maqalah*	*la*	*Einstein*_*i*_	*hwwa*_*i*_	*ɡara-ha?*	
		which	article	of Einstein_*i*_	he_*i*_	read-it?		
		‘∗Which article of Einstein_*i*_ did he_i_ read?'						
	b.	*l- maqalah*	*la*	*Einstein*_*i*_	*yalli*	*hwwa*_*i*_	*ɡara-ha?*	
		the-article	of	Einstein_*i*_	Rel	he_*i*_	read-it?	
		' The article of Einstein_*i*_ that he_*i*_ read ' (Abu Helal 2021a: p.12)						

Again, the ungrammaticality of (28.b) is unexpected if we assume that the *yalli* relative involves a raising syntax based on A-bar movement with reconstruction effects. A head NP raising structure would wrongly rule in the ungrammatical sentence in (28.b) by inducing a condition A bleeding effect. Similarly, a head NP raising structure would wrongly rule out the grammatical sentence in (29.b) by inducing a condition C violating effect.

Based on the facts (27–28), we can safely conclude that the *yalli* relative may have a head external matching structure in addition to the analysis in (26).

### 3.2 On the amount interpretation of the yalli relative in A-and G-superlatives

This subsection argues for two facts about the *yalli* relative clause with the low reading in A- and G-superlatives. First, the *yalli* relative has an amount interpretation (i.e., it denotes a predicate of degrees of type <dt>). Second, the superlative morpheme that modifies the *yalli* relative clause is not internally represented within the *yalli* relative clause. These two facts suggest that the *yalli* relative clause with the low reading involves a relation of degree operator-variable dependency that is derived via raising of a null degree operator.^19^

First, we observe that the emergence of low reading in A-and G superlatives is sensitive to constraints on the degree operator-variable dependency formed by movement. The fact that the low reading is blocked under locality and islandhood conditions of degree movement strongly suggests that the *yalli* relative involves predication of degrees based on a degree operator movement.

One constraint is what Heim (2000) called Kennedey's Generalization after observations in Kennedy (1999) defined in (30).

**Table:** 

(30)	**The Heim-Kennedy Constraint:**	
	If the scope of a quantificational DP contains the trace of a DegP, it also contains that DegP itself	
		(Heim 2000:27)

This generalization restricts the scopal behavior of degree operator in such a way that it cannot scope over other quantificational operators which c-command the degree variable that is quantified over by the degree operator. Consider the A-and G-superlatives in (31) and (32). In the two structures, the low reading is absent: the two structures are unambiguous and the only reading they have is the high reading. Clearly, the presence of the quantificational NP *kul wahed* ‘every one’ blocks the low reading in (31) and (32) is. That is, the dependency between the degree operator and its associate scalar term which is expressed by the lower copy of the movement deletion (i.e., d-long article) is interrupted by an intervening QP. By contrast, the degree operator-variable dependency that gives rise to the high reading has no such intervening effect and hence the high reading obtains in (31) and (32).

**Table:** 

(31)	*ʕali*	*ɡara*	*l-maqalah*	*l-ʔtˤwal/*	*ʔtˤwal*	*maqalah*
	Ali	read	the-article	the-longest/	longest	article
	*yalli*	*kull*	*waħ*əd *ħaka*	*ʔinnu*	*Einstein*	*katab-ha*
	Rel	every one	said	that	Einstein	wrote-it
	Literal: ' Ali read the article the longest/longest article that everyone said that Einstein wrote. '					
	≈: ' Ali read the longest article that everyone said that Einstein wrote. '					
	#**Low Reading:**					
	The longest article λd [_CP_ [that ∀x [ person (x) → x said that Einstein d-long article]					
	' The x such that everyone said that x was the longest paper written by Einstein'					
	**High Reading:**					
	The longest article λd [_CP_ d-long article [ that ∀x [ person (x) → x said that Einstein]					
	'The longest paper about which everyone said that Einstein had written it '

**Table:** 

(32)	*ʕali*	*ɡara*	*l-maqalah*	*l-ʔtˤwal/*	*ʔtˤwal*	*maqalah*
	Ali	read	the-article	the-longest/	longest	article
	*yalli*	*l-ustaz*	*ħaka*	*ʔinnu*	*kull waħ*əd	*katab-ha*
	Rel	the-teacher	said	that	everyone	wrote-it
	Literal: ' Ali read the article the longest/longest article that the teacher said that everyone wrote it.'					
	≈: ' Ali read the longest article that the teacher said that everyone wrote it. '					
	#**Low Reading:**					
	The longest article λd [_CP_ [ that the teacher said that ∀x [ person (x) → x wrote d-long article]					
	' The x such that the teacher said that x was the longest paper written by everyone '					
	**High Reading:**					
	The longest article λd [_CP_ d-long article [ that the teacher said that ∀x [ person (x) → x wrote d]					
	' The longest paper about which the teacher said that everyone had written it '					

As such, we predict that syntactic islands such as adjuncts and wh-constituents block the low reading as well. This is indeed the case. In (33), for example, the wh- island blocks the low reading.

**Table:** 

(33)	*ʕali*	*ɡara*	*l-maqalah*	*l-ʔtˤwal/*	*ʔtˤwal*	*maqalah*
	Ali	read	the-article	the-longest/	longest	article
	yalli	l-ustaz	ħaka	mata	Einstein	katab-ha
	Rel	the-teacher	said	when	Einstein	wrote-it
	Literal: ' Ali read the article the longest/longest article that the teacher said when Einstein wrote. '					
	≈: ' Ali read the longest article that the teacher said when Einstein wrote. '					
	#**Low Reading:**					
	The longest article λd [_CP_ [ that the teacher said when Einstein wrote d-long article]					
	' The x such that the teacher said when Einstein wrote the longest article x '					
	**High Reading:**					
	The longest article λd [_CP_ d-long article [ that the teacher said when Einstein wrote d- long article ]					
	' The longest paper about which the teacher said when Einstein wrote it '					

Similarly, the adjunct island blocks the low reading as in (34).

**Table:** 

(34)	a.	*ʕali*	*ɡara*	*l-maqalah*	*l-ʔtˤwal/*	*ʔtˤwal*	*maqalah*			
		Ali	read	the-article	the-longest/	longest	article			
		yalli	l-ustaz	ħaka	ʔinnu	Max	kayyaf	ʕaʃaan	Einstein	katab-ha
		Rel	the-teacher	said	that	Max pleased	because	Einstein	wrote-it	
		Literal: ' Ali read the article the longest/longest article that the teacher said that Max was pleased because Einstein wrote it. '								
		≈:' Ali read the longest article that the teacher said that Max was pleased because Einstein wrote it. '								
		#**Low Reading:**								
		The longest article λd [_CP_ [ (yalli the teacher said that Max was pleased because Einstein wrote d-long article]								
		' The x such that the teacher said that Max was pleased because Einstein wrote x '								
		**High Reading:**								
		The longest article λd [_CP_ d-long article [ (ya)illi the teacher said that Max was pleased because Einstein wrote d-long article]								
		' The longest paper about which the teacher said that Max was pleased because Einstein wrote it '								

These facts show that the low reading in the A-and G-superlative is sensitive to the locality conditions that constrain the formation of degree operator-variable dependencies. We conclude that the *(ya)illi* relative clause with the low reading has an amount reading based on quantification of degrees and the low reading of the *(ya)illi* relative involves a dependency that is subject to constraints on degree movement.

Second, the degree operator-variable dependency detected in the *(ya)illi* relative clause in A- and G-superlatives doesn't involve an internal representation of the external superlative operator. This degree operator is base-generated in the external position of the relativized head NP and it is not interpreted as part of the internal representation of the external NP derived by movement deletion within the *(ya)illi* relative clause.^20^ Evidence for this claim comes from the fact that the superlative morpheme never scopes beyond its surface position: it has scope at or above the surface position of the relativized external NP head. Consider the following sentence.

**Table:** 

(35)	**Scenario 1**
	Robert is teaching an advanced physics seminar. Ali, Ad and Ted are enrolled students into the seminar. Each student expressed an interest in reading a certain number of articles that were authored by Einstein as follows.
	Ali wants to read 3 articles that Einstein wrote and no one else reads 3 articles Ad wants to read 4 articles that Einstein wrote Ted wants to read 5 articles that Einstein wrote
	*Robert ɡaabal l- tˤaalib l-ʔktar/ʔktar tˤaalib yalli bidu yɡara maqalalit la Einstein* Robert met the student the most/most student Rel wanted read article of Einstein ‘ Robert met with the student who wants to read the most articles.’

In the context that Robert met with Ali, the superlative in (35) is false. It can only be true if the superlative operator *ʔktar* ‘most’ is interpreted in the scope of the intensional operator *bidu* ‘want’ as represented by the infelicitous structure (36).

**Table:** 

(36)	# Robert met with the unique x such that x is a student and ∀w’ compatible with x's desires in w such that ∃d [ x reads d-many articles of Einstein in w’] & ∀Q ∈ C [ Q ≠ (λd’. x reads d’-many articles of Einstein in w’) → ¬ Q(d)]]

Therefore, the superlative structure in (36) only has the logical form in (37) where the superlative operator has scope at or above the surface position of the relativized NP head *the student the most.*^21^

**Table:** 

(37)	Robert met with the unique x such that ∃d [ x is a student and ∀w’ compatible with x's desires in w such that [ x reads d-many articles of Einstein in w’] & ∀Q ∈ C [ Q ≠ (λd’. x reads d’-many articles of Einstein in w’) → ¬ Q(d)]]

These facts suggest that the superlative operator is base-generated in its surface position in the external head NP and it is never represented within the *yalli* relative clause in A-superlatives. The overall conclusion of this section is that the *yalli* relative clause with the low reading has amount reading derived by the movement of a null degree operator that creates degree operator-variable dependency.

### 3.3 Late merger

Under the copy theory of movement (Chomsky 1977, 1995), the grammar interprets copies of movement chain in different positions.^22^ As a consequence, a reconstruction effect arises in (38) where the lower copy of the A-bar moving element containing the R-expression *John*_*i*_ is interpreted within the c-command domain of the R-expression's referential pronoun *he*, resulting into condition C violation.

**Table:** 

(38)	∗[Which argument that John_i_ is a genius] did he_i_ believe ? (Fox 1999:164 as cited in Takahashi and Hulsey 2009: 391)

Such a reconstruction effect can be found in A-movement as well. For example, the structure in (39) has a bound-variable reading in which the pronoun that is dominated by the lower copy of the A- moving DP that dominates the pronoun *his*_*i*_ is interpreted in the scope of its quantificational binder *every professor* in LF.

**Table:** 

(39)	[Someone from his_i_ class] seems to [every professor]_i_ to be a genius [Someone from his_i_class]

Based on the data in (42) and (43), it can be concluded that movement, be it A-or A-bar movement, may leave fully interpretable copies and hence it may trigger reconstruction effects.

Nevertheless, it has been observed that A- and A-bar movement differ with respect to Condition C. Unlike the A-bar movement structure in (40) which has Condition C effect, the A-movement structure in (41) doesn't induce such an effect.

**Table:** 

(40)	∗Which argument that John_i_ is a genius did he_i_ believe ?

**Table:** 

(41)	a.	[The claim that John_i_ was asleep] seems to him_i_ to be correct
	b.	[Every argument that John_i_ is a genius] seems to him_i_ to be flawless.

The grammaticality of (41) is unexpected under the copy theory of movement. If the A-moving expression may leave an interpretable copy (i.e., as evidenced in (43)), a condition C effect is predicted. Contrary to the fact, the sentences in (41) are grammatical and it appears to be the case A-movement bleeds Condition C.

The standard solution to this asymmetry between A- and A-bar movement is to assume that the expression dominating the R-expression in (41) undergoes Late Merger. Such a limited countercyclic merger removes the DP containing the R-expression *John* from the c-command domain of the third person singular masculine pronoun. As for (40), independent properties of the grammar may prevent the application of the late merger of the sentential restrictor of the A-moving wh-phrase and hence a reconstruction effect arises (Lebeaux 1988; Fox 2002; Takahashi and Hulsey 2009, Santon, 2016; among many others).

On the LF interpretability approach (Fox 2002), late merger is applicable as long as the output is semantically interpretable. Fox's semantic approach nicely accounts for the fact that adjuncts, unlike complements, may be introduced countercyclically into structure.^23^ Accordingly, the reason why (42.a) is grammatical is that the relative clause ‘that John_i_ made’ undergoes late merger and such an operation yields a semantically interpretable output. Assume that the relative clause ‘that John_i_ made’ denotes a predicate of individuals of type <et>. Assume further that the DP ‘which argument’ undergoes QRing. The relative clause, ‘that John_i_ made’, can compose with the denotation of argument via predicate modification.

**Table:** 

(42)	a.	Which argument [that John_*i*_ made] did he_*i*_ believe?	
	b.	∗Which argument [that John_*i*_ is a genius] did he_*i*_ believe?	
			(Fox 1999: 164)

The late merger of the complement ‘that John_*i*_ is a genius’ in (45.b), on the other hand, fails to produce a compositionally interpretable representation and hence late merger should not be an option for cases like (42.b). Let us see how the late merger of the complement ‘that John_*i*_ is a genius’ doesn't yield a compositionally interpretable output.

On Fox's (2002) approach, the trace Conversation mechanism interprets the copies of a movement chain in the semantics. This mechanism converts movement copies into definite descriptions of type e using two derivational steps: (i) variable insertion turns (Det) Pred into (Det) [ Pred λy(y = x)] where the Pred and the inserted variable [λy(y = x], being predicates of the same type (i.e., type <et>), combines via Predicate Modification as represented in (43).

**Table:** 

(43)	(Det) [λy [ Pred (y) & (y = x)]

(ii) determiner replacement where the Det is replaced by a definite description operator that applies to the predicate that is introduced by predicate modification to yield (44), which is an expression of type e.

**Table:** 

(44)	ιy [ Pred (y) & (y = x)].

Given this assumption, we can see how the complement in (42.b) may not undergo late merger. Assume that the lower copy of the wh-phrase ‘which argument’ in (42.b) is of type <t,et> .^24^ Then, there would be no way to apply the trace conversation mechanism: the DP ‘which argument’ that stands for Pred, which is of type <t,et> and the predicate λy(y = x) which is of type <et> cannot compose intersectively via predicate modification. As a result, the lower copy remains non-compositionally interpretable (Takahashi and Hulsey 2009: 396–397).

The LF interpretability approach (Fox 2002) may extend to other environments where late merger produces semantically interpretable representations: wholesale late merger WLA. In this environment, an operator/determiner may undergo QRing and its restrictor saturates its first argument via late merger provided that no other independent properties in the grammar rules out this possibility (Fox and Nissenbaum 1999, Bhatt and Pancheva 2004; Takahashi and Hulsey 2009, Santon, 2016).

To illustrate, Fox and Nissenbaum (2000) observed that adjunct extraposition bleeds Condition C as shown in (45).

**Table:** 

(45)	a.	I gave him_i_ a picture from John_i_'s collection yesterday	
	b.	I gave him_i_ a picture yesterday [ from John_i_'s collection].	
			(Fox and Nissenbaum, 2000: 139)

They analyzed the fact in (45) as follows. The DP ‘a picture’ undergoes rightward QRing across the adverbial yesterday. The movement forms a chain in which the DP is pronounced in the tail. Then the adjunct merges late to compose with the restrictor of the DP ‘argument’ as represented in (46).

**Table:** 

In this way, late merger removes the R-expression dominated by the relative clause from the c-command domain of its co-referential pronoun and Condition C can be circumvented by extraposition of adjuncts. Notice that the tree (46) is a semantically interpretable output. Interestingly, Bhatt & Pancheva (2004) observed that comparative constructions also induce a Condition C bleeding effects by extraposition of comparative complements as in (47).

**Table:** 

(47)	a.	?? I will tell him_i_ a sillier rumor about Ann [ than Mary told John_i_]
	b.	I will tell him_i_ a sillier rumor about Ann tomorrow [ than Mary told John_i_]

Bhatt and Pancheva (2004) accounted for Condition C bleeding effect in (48) by postulating a late merger solution: the –er operator undergoes rightward QRing and the comparative complement [ than Mary told John_i_] merges late to compose as the restrictor of the degree operator as schematized in (48).

**Table:** 

Again, the late merger of the comparative complement removes the R-expression dominated by the comparative complement from the c-command domain of its co-referential pronoun and Condition C can be circumvented by extraposition of comparative complements. The tree in (48) is semantically interpretable as well.

Back to the asymmetry between A- and A-bar movement w.r.t. Condition C, Takahashi and Hulsey (2009) proposed that the reason why Condition C is circumvented in A-movement in (49) is that the structure involves the wholesale merger of the clause ‘[that John_*i*_ is a genius] as schematized in (50):

**Table:** 

(49)	a.	Every argument [that John_*i*_ is a genius] seems to him_*i*_ to be flawless.	
	b.	∗ Which argument [that John_*i*_ is a genius] did he_*i*_ believe?	
			(Takahashi and Hulsey (2009): 164)

**Table:** 

As shown in (50), the restrictor dominating the R-expression may undergo wholesale late merger with the QRed determiner into a position that is higher than the c-command domain of the co-referential pronoun, but still lower that the Case assigning functional head T.^25^ In this way, the whole DP including the restrictor can be assigned nominative Case by entering into Goal-Probe Agree with T. In the same time, Condition C effect can be reversed by removing the R-expression out of the c-command domain of its co-referential pronoun.

In the contrary, Takahashi and Hulsey (2009) argued that the wholesale late merger of the complement [that John_*i*_ is a genius] is blocked in A-bar movement in (51), repeated from (49.b) and therefore a Condition C effect arises.

**Table:** 

(51)	∗Which argument that John_i_ is a genius did he_i_ believe ?

They proposed that it is Case Filter that prohibits this operation. On the assumption that the accusative Case marking assignment of the wh-phrase ‘which’ along with its sentential complement [argument that John_*i*_ is a genius]’ is achieved structurally by a Goal-Probe Agree operation based on c-command relation with the Case assigning functional head v (Chomsky, 1998), the restrictor dominating the R-expression cannot undergo wholesale late merger since this operation would locate the restrictor outside the c-command domain of v. To get Case-marked, the whole DP ‘which argument [that John_*i*_ is a genius]’ needs to be base-generated within the complement of the VP and the R-expression dominated by the restrictor would come within the c-command domain of a co-referential pronoun resulting in a condition C violation as schematized in (52).

**Table:** 

We saw in the preceding section that the A- and G- superlative structures show a similar asymmetrical behavior as exemplified in (53).

**Table:** 

(53)	*l-ustaz*	*ħaka*	*l-u*_*i*_	*yiɡadim*	*l-maqalah*	*l-ʔtˤwal*	*/∗ʔtˤwal*	*maqalah*
	The-teacher	told	to-him	present	the-article	the-longest/	∗longest	article
	yalli	ʕali_i_	ɡaraʔ-ha					
	Rel	Ali_*i*_	read-it					
	‘The teacher told him_i_ to present the longest article that Ali_i_ read.’							

The straightforward solution to the asymmetry in (53) is to assume that A-superlative involves the late merger of the relative clause *yalli Ali*_*i*_ *qaraʔ-ha* ‘yalli Ali read it’ as high as the VP projection. Such an operation removes the R-expression Ali_i_ that is dominated by the *yalli* relative clause out of the c-command domain of its co-referential pronoun. For an independent reason (i.e., Case filter), the grammar prohibits the application of the late merger of the *yalli* relative clause in the G-superlative. As a result, the structure is ungrammatical due to Condition C violation.

We will address this question by making one more assumption made in Bhatt and Pancheva (2004, 19): a high-attachment site in A-superlative in (53) becomes accessible whenever the structure has a new scopal reading resulting from having the-est scoping over the VP *ħaka lu*_*i*_ ‘told to him_*i*_’. The fact that the A-superlative structure in (53) has a new scopal reading where the comparative operator –est takes scope over the VP *ħakalu*_*i*_ ‘told to him_*i*_’ makes available a high-attachment site for the *yalli* relative clause. Such reading holds true if the teacher told Ali to read the article x to a degree d and there is no other article among the ones that Ali read which is as long or longer than d. This reading corresponds to the structure in which the –est operator takes scope over the VP with its comparison class being expressed by the *yalli* relative clause.

**Table:** 

(54)	–est > tell: The teacher told him: present the d-article where there is no other d’- long article that Ali read such that d > d’

A reading where the VP *ħaka lu*_*i*_ ‘told to him_*i*_’ scopes over the superlative –est is not available. Such a reading says that the teacher told Ali “read the d-long article that is longer than all other articles in the comparison set”.

**Table:** 

(55)	tell > -est: the teacher told him: present d-long article; there is no other d’-long article that Ali read such that d > d’.

As expected, the A- and G-superlative structures differ with respect to Condition C. Consider, again (56). Unlike the A-superlative, the G-superlative induces a Condition C effect since the *yalli* clause contains an R-expression that sands in a co-referential relation with a c-commanding pronoun.

**Table:** 

(56)	*l-ustaz*	*ħaka*	*l-u*_*i*_	*yiɡadim*	*l-maqalah*	*l-ʔtˤwal/*	*∗ʔtˤwal*	*maqalah*
	The-teacher	told	to-him	present	the-article	the-longest/	∗longest	article
	yalli	ʕali_i_	ɡaraʔ-ha					
	Rel	Ali	read-it					
	‘The teacher told him_i_ to present the longest article that Ali_i_ read.’							

Again, the condition C bleeding effect in the A-superlative suggests that there should be a high attachment site for the merger of the *yalli* relative clause outside the c-command domain of the pronoun in the A-superlative structure. This site is not available in the G-superlative structure. As a result, the G-superlative structure is ruled out due to condition C violation.

In principle, the superlative constructions in (56) are structurally ambiguous between a high-attachment structure that is located outside the c-command domain of the co-referential pronoun and the low-attachment structure that falls within the pronoun's c-command domain. Both structures are superficially identical. We will assume, following Fox (2002) and Bhatt and Pancheva (2004) that the high attachment structure is blocked due to a parsing preference in which the low attachment structure is favored over the high attachment one. Given this parsing preference condition, a high attachment site for the *yalli* relative clause is excluded in the G-superlative in (56.b) and hence a condition C violation arises.

For some reason, this parsing preference condition doesn't obtain in A-superlative so that we get a superlative with a Condition C bleeding effect. A question then arises: why such a preference condition doesn't extend to the G-superlative structure in (56) where a high-attachment site is inaccessible resulting in a Condition C violating effect. Put it differently, what licenses the higher attachment site in A-superlative structure to the exclusion of the G-superlative structure.

The answer to this question is as follows. Because the –est quantifier is allowed to QR followed by the wholesale late merger of the *yalli* relative in A-superlatives, a new scopal reading arises and such a reading licenses a high attachment site besides a low attachment one. Therefore, the parsing preference condition does not obtain in A-superlatives. When it comes to G-superlatives, the wholesale late merger of the *yalli* relative is blocked by the Case requirement, resulting in the absence of a higher scopal reading that may license a higher attachment site. This makes G-superlatives subject to the parsing preference condition.

## 4 The analysis

### 4.1 Syntax-semantics of (ya)illi relative clause in A-superlatives

So far, we argued for two facts about the *yalli* relative clause in (57): (i) the *yalli* relative clause has a head external structure (e.i., matching) and (ii) the *yalli* relative clause with the low reading is an amount relative clause derived by degree operator movement.

**Table:** 

(57)	*ʕali*	*ɡara*	*l-maqalah*	*l-ʔtˤwal/*	*ʔtˤwal*	*maqalah*
	Ali	read	the-article	the-longest/	longest	article
	yalli	Aħmad	ħaka	ʔinnu	Einstein	katab-ha
	Rel	Ahmad	said	that	Einstein	wrote-it
	Literal: ' Ali read the article the longest/longest article that Ahmad said that Einstein wrote. '					
	≈: 'Ali read the longest article that Ahmad said that Einstein wrote. '					

The standard view of Grosu and Landman (1998), among many others, is that degree relative clauses involve a raising syntax where the DegP raises along with the head NP into the left periphery of the CP and then the head noun raises out of the CP, as schematized in (58).

**Table:** 

Since evidence points to the fact that the *yalli* relative clause has a head external structure and such a structure has an amount reading based on the movement of an internal null degree operator, our analysis makes use of a matching structure along the lines of Szczegielniak (2012). Accordingly, the degree reading of the relative clause can be derived under matching using the following steps: first, the head NP is base-generated in its surface structure outside the relative clause with the degree phrase being located in its specifier position. Second, the internal representation of the head NP undergoes movement deletion into an internal Spec-Topic position where it deletes under ellipsis of the sort of Topic drop (Huang, 1984). Finally, the DegP raises outside the relative clause with the consequence of creating a degree abstract that creates a predicate of degrees which corresponds to the amount reading of structure.

We will extend a matching analysis to the degree *yalli* relative clauses in A-and G-superlatives with the low reading along the lines of Szczegielniak (2012). Since evidence speaks against the reconstruction of the –est operator within the *yalli* relative clause, we will assume that the degree operator-variable dependency is achieved by the raising of a null degree operator from its base generated position in the internal Spec-NP head into the external Spec- DP of the *yalli* relative. On this analysis, the *yalli* relative clause^26^ in (57) will have the following syntax- semantics as schematized in the syntactic and semantic trees in (59) and (60), respectively.

**Table:**

**Table:** 

The syntax- semantics of the *yalli* relative involves three major sequential steps: first, the internal NP head along with its null degree operator, which is base generated in the Spec-AP of the internal NP, raises into an internal Spec-Topic position. It then creates two copies of the internal NP of type e. Second, the null degree operator undergoes movement into the external Spec-DP of the relative clause leaving behind a trace of typed that saturates the degree argument of the scalar adjective of the two copies of the internal NP. Third, the internal NP deletes under identity via an ellipsis-based movement deletion.

### 4.2 The low reading derived

Recall that A- and G- superlative structures in (61) are ambiguous between the low/high readings (Bhatt 2002; Hulsey and Sauerland 2006).

**Table:** 

(61)	*ʕali*	*ɡara*	*l-maqalah*	*l-ʔtˤwal/*	*ʔtˤwal*	*maqalah*
	Ali	read	the-article	the-longest/	longest	article
	yalli	Aħmad	ħaka	ʔinnu	Einstein	katab-ha
	Rel	Ahmad	said	that	Einstein	wrote-it
	Literal: ' Ali read the article the longest/longest article that Ahmad said that Einstein wrote. '					
	≈: 'Ali read the longest article that Ahmad said that Einstein wrote. '					
	**High Reading:**					
	Ali read the longest paper about which Ahmad said that Einstein had written it.					
	**Low Reading:**					
	Ahmad read the x such that Ahmad said that x was the longest paper written by Einstein.					

In what follows, we propose an analysis based on the late merger of the *yalli* relative clause that derives a semantically interpretable output for the low reading of the *yalli* relative in (61).Recall that under the low reading, the A-superlative is ambiguous between the absolute/relative interpretations as in (62).

**Table:** 

(62)	**Low reading**		
	**(absolute)**	**✓ A-superlative**	**✓ G-superlative**
	It is true iff Ahmad read an article that is long to a degree to which no other article in Ali's saying -worlds is long.		

On a late merger analysis and following a head-external analysis (Heim 1999, Szabolcsi, 1986), deriving the low reading of the *(ya)illi* relative clause with an absolute interpretation involves the following steps: first, the ⟦ -est C ⟧ moves minimally within the DP in the A- superlative the *article longest* and it creates a degree abstract over the degree d in its base generation position.

**Table:** 

Since the sister of ⟦ -est C ⟧ in (63) denotes the predicate of degrees [λd. PRO_x_ is d-long article], each member of ⟦ C ⟧ is a predicate of degrees of type <dt>. On this way, the comparison class complement ⟦ -est C ⟧ denotes predicates of predicates of degrees of type <dt, t> which stands for the comparison class complement of the first argument of the ⟦ -est ⟧ operator G (i.e., ⟦ -est ⟧ = λG_<dt,t>_ λP_<dt>_. ∃d [ P(d) ∧ ∀Q ∈ G [ Q ≠ P→ ¬ (Q(D))]). Intuitively, this set of predicates of degrees include the set that PRO_x_ and other relevant articles are long to.^27^

Second, we propose that the *(ya)illi* relative clause composes countercyclically via Late Merger: the *(ya)illi* relative clause composes with the comparison class complement G via predicate modification. To enable this operation, the denotation of (*ya)illi* relative clause should be type- lifted from the compositionally derived predicate of degrees <d,t> into a predicate of predicates of degrees of type <dt, t>. To achieve this, we let the SHIFTER operator in (64) operate on the denotation of the *(ya)illi* relative clause, which takes the predicate of degrees denoted by the *(ya)illi* relative clause into a predicate of upper-bound degree predicates.

**Table:** 

(64)	SHIFTER_<dt> → <dt,t>_ =: λD_<dt>_ λD’_<dt> ._ ∃d’ [D(d’) & D’ = λd’’. d’’≤ d’]	
		(Romero 2011: 92)

The *(ya)illi* relative clause in (64) denotes the predicate of degrees is type-shifted into a predicate of predicates of degrees by applying the SHIFTER operator in (65).

**Table:** 

(65)	SHIFTER_<dt> → <dt,t>_ =: λD_<dt>_ λD’_<dt> ._ ∃d’ [D(d’) & D’ = λd’’. d’’≤ d’] ([λd. Ali said that Einstein wrote [ιx. x is d-long y]]) = : λD’_<dt,t> ._ ∃d’ [Ali said that λw’ Einstein wrote a d’-long article in w'in w’] & D’ = λd’’.d’’≤ d’]

At this point, the *(ya)illi* relative clause undergoes late merger to compose with the denotation of ⟦ C ⟧ via predicate modification.

**Table:** 

The comparison class complement of the –est operator will have the following denotation that is the result of composing the late merged *(ya)illi* relative clause in (65) with the denotation of ⟦ C ⟧

**Table:** 

(67)	⟦ XP ⟧ = : ⟦ C ⟧ ∩ [λD’_<dt,t> ._ ∃d’ [Ali said that λw’ Einstein wrote a d’-long article in w’] & D’ = λd’’. d’’≤ d’]

Finally, the LF representation that delivers the truth conditions of the low reading of the *(ya)illi* relative with an absolute interpretation in A- is given in (68).^28^

**Table:** 

(68)	Ali read the unique x: ∃d [ article (x) & long (x,d) & ∀Q ∈ [ C ∩ [λD’_<dt> ._ ∃d’ [Ahmad said that λw’ Einstein wrote a d’-long article in w’] & D’ = λd’’. d’’≤ d’] [ Q ≠ (λd’. x is a d’-long article) → ¬ Q(d)]

When it comes to the relative low reading, an asymmetry between the two types of superlatives arises: while the A-superlative may have a relative reading, such a reading is blocked in G-superlatives.

**Table:** 

(69)	**Low Reading**		
	**(relative)**	**✓ A-superlative**	✗ **G-superlative**
	It is true iff Ali read an article that is long to a degree such that no other individual read an article in Ahmad's saying -worlds and it is long to that degree.		

Deriving the low reading of the *yalli* relative in the A-superlative with relative interpretation involves the following steps. First, the DegP ⟦ -est C ⟧ undergoes QRing into the scope position of VP in the A-superlative in (70) the *Ali read the longest article* and it creates a degree abstract over the degree d in its base generation position.

**Table:** 

The sister of ⟦ -est C ⟧ denotes a predicate of degrees [λd. Ali read a d-long article]. Therefore, the members of ⟦ C ⟧ are the predicates of degrees such that Ali and other article-readers read a d-long article. On this way, the comparison class complement ⟦ -est C ⟧ denotes predicates of predicates of degrees of type <dt, t> which represents the comparison class complement of the first argument of the ⟦ -est ⟧ operator G.

We propose that the *yalli* relative clause undergoes late merger to compose with the comparison class complement C via predicate modification as represented. The type shifted *yalli* relative clause with the denotation. λD’_<dt> ._ ∃d’ [Ahmad said that λw’ Einstein wrote [ιx. x is d’-long y ] in w’] & D’ = λd’’. d’’≤ d’] composes with ⟦ C ⟧ intersectively as represented in (71).

**Table:** 

(71)	a.	⟦ XP ⟧ = : ⟦ C ⟧ ∩ [λD’_<dt> ._ ∃d’ [Ahmad said that λw’ Einstein wrote a d’-long article in w’ & D’ = λd’’. d’’≤ d’]
	b.	

The LF representation in (72) expresses the truth conditions of the relative low reading of A-superlative.

**Table:** 

(72)	∃d [∃x [ article (x) & long (x,d) & ali read (x,d)] &∀Q ∈ [ C ∩ [λD’_<dt> ._ ∃d’ [Ahmad said that λw’ Einstein wrote a d’-long article in w’] & D’ = λd’’. d’’≤ d’] [ Q ≠ (λd’. λd. ahmad read a d-long article) → ¬ Q(d)]

We assume that the whole DP *the longest paper* along with its nominal modifier the *yalli* relative clause *YALLI Ahmad said that Einstein wrote* requires Case. Since the superlative head undergoes QRing into a Case-assigning position (i.e., within the c-command domain of the functional v head), the *yalli* relative can be introduced at this position to receive Case.

So what blocks the relative reading of G-superlatives with the low reading?! Recall that under the late merger analysis, the relative low reading of the G-superlative is derived through the DegP ⟦ -est C ⟧ QRing into Spec-VP followed by the late merger of the *yalli* relative clause to compose with the denotation of the comparison class complement ⟦ C ⟧ via predicate modification. The unavailability of this reading is well predicted under the current analysis: since the NP head along with its *yalli* relative clause reading requires Genitive Case which is assigned by the Case assigning functional head D^0^,^29^ the *yalli* relative clause cannot be introduced outside the c-command domain of D^0^ (i.e., higher than the DP at the VP level). In this way, deriving the relative low reading of G-superlative is blocked by Case Filter as shown in (73).

**Table:** 

Our analysis makes one more correct prediction: it explains the asymmetry between A-superlative and G-superlative with respect to Condition C as exemplified in (74), repeated from (53).

**Table:** 

(74)	*l-ustaz*	*ħaka*	*l-u*_*i*_	*yiɡadim*	*l-maqalah*	*l-ʔtˤwal/*	*∗ʔtˤwal*	*maqalah*
	The-teacher	told	to-him	present	the-article	the-longest/	∗longest	article
	yalli	ʕali_i_	qaraʔ-ha					
	Rel	Ali_*i*_	read-it					
	‘The teacher told him_i_ to present the longest article that Ali_i_ read.’							

Recall that the A-superlative in (74) has a reading in which the comparative operator –est takes scope over the VP *‘told-him’*. Such a reading holds true if the teacher told Ali to read the article x to a degree d and there is no other article among the ones that Ali read which is as long or longer than d. A late merger analysis where the est-operator undergoes QRing as high as the VP *‘told-him’* followed by the late merger of the *yalli* relative clause can derive this new scopal reading and it also removes the R-expression Ali_i_ that is dominated by the *yalli* relative clause out of the c-command domain of its co-referential pronoun. Therefore, the A-superlative in (74) gives rise to a Condition C bleeding effect. Notice that this operation is not blocked by Case filter: the superlative operator QRs into a Case assigning position (i.e., within the c-command domain of the accusative Case assigning head, the matrix v and the *yalli* relative, which also demands accusative Case, is introduced in this Case assigning position.

**Table:** 

As for the ungrammatical G-superlative in (74.b), since the late merger of the *yalli* relative as high as the matrix VP is blocked by the need of the *yalli* relative clause to be Case-marked at the lower DP level, a condition C effect is inevitable.

## 5 Conclusion

In this paper, we introduced a puzzle related to an asymmetry between the so-called (A)ttributive and (G)enitive superlatives that modify *yalli* relatives in Jordanian Arabic: while the A-superlative is ambiguous between the absolute and relative readings under the low reading of the *yalli* relative (Bhatt 2002), the G-superlative is unambiguous and the only reading it has is the absolute reading.

To resolve the puzzle, we proposed a compositional analysis that explains the asymmetry between these two modes of superlative modification in *yalli* relatives. Our analysis is based on the following well-motivated working assumptions that were used to derive the low reading of the *yalli* relative clause: (i) the *yalli* relative clause undergoes late merger to compose with the comparison class complement of the QRed operator via predicate modification. (ii) Such composition may take place at the DP level to form the LF associated with the absolute low reading of the A- and G superlative and at the VP level to form the LF associated with the relative low reading in A- superlatives. (iii) Since late merger is controlled by Case filter (Takahashi and Hulsey 2009: 164), the relative low reading of G-superlatives is blocked because the *yalli* relative in this structure cannot be introduced outside the c-command domain of the genitive Case assigning head within the DP. And finally, (iv) such a Case requirement explains the asymmetry between the A- and G-superlatives with respect to Condition C.

## Declarations

### Author contribution statement

Abdel-Rahman Abu Helal: Conceived and designed the experiments; Performed the experiments; Analyzed and interpreted the data; Contributed reagents, materials, analysis tools or data; Wrote the paper.

### Funding statement

This work was supported by the Deanship of Scientific Research at Al-Zaytoonah University of Jordan [grant number QF21/1803-3.0].

### Data availability statement

Data will be made available on request.

### Declaration of interests statement

The authors declare no conflict of interest.

### Additional information

No additional information is available for this paper.

## Footnotes

3 We assume the following language L with the following basic ontological semantic type system: e ∊ De (the domain of entities); t ∊ Di (the domain of times); d ∊ D_d_ (the domain of degrees); the set of truth values {TRUE, FALSE} and D_⟨α, β⟩_ = $D_{\beta }^{D\text{α}}$ along with the interpretation function ⟦ ⟧ which is a function that assigns interpretations to the lexical elements in composition.

4 We may take measure phrases as generalized quantifiers that denote predicates of predicates of degrees of type <dt,t>. Then, they can undergo Quantifier Raising and degree abstraction in such a way that the degree argument is quantified over just as other degree modifiers that function as degree quantifiers (Schwartzschild and Wilkinson 2002).

5 Another lexical entry of the superlative operator exists (i). It is represented as a 3-place predicate that applies to three arguments individual-denoting objects, a predicate of individuals and a gradable adjective. The application of this operator yields the truth conditions in which there is a degree d such that the individual x has a property P to that degree and all the other individuals in the contextually determined comparison class G don't have the property to d (See Romero 2011 for an assessment of the superlative as a 2-place and 3-place predicate). (i) ⟦ -est ⟧ = λG_<e,t>_ λP_<dt,t>_ λx. ∃d [ P(d) (x) ∧ ∀y ∈ G [ y≠ x→ ¬ (P(d) (y))]

6 On a head-internal analysis (Heim 1985; Sharvit and Stateva 2002), the ambiguity is contextually-determined: the superlative operator involves a contextual variable C that is valued by context in such a way that it denotes a set of contextually-determined articles in the absolute reading and a set of articles read by contextually-determined individuals. The superlative operator undergoes minimal QR within the DP along with its restrictor C that is valued by context to derive the ambiguity. (i) a. **Absolute Reading:** Ali read the unique x such that ∃d [ x is an article and x    is d-long ∧ ∀Q ∈ C [ Q ≠ (λd’.x is a d’-long article) → ¬Q(d)]]  b. **Relative Reading:** ∃d [∃x[ x is an article and x is d-long and Ali read x ] ∧ ∀Q ∈ C [ Q ≠ (λd’. Ali read a d’-long article) → ¬ Q(d)]

7 meaning that the members of the comparison class C must have the same domain as the denotation of [-est C]'s sister in LF.

8 This prescriptive rule of superlative formation only applies to a set of adjectives with triple roots (i.e., C_1_C_2_C_3_) which are gradable, affirmative and fully agreeing properties with the exception of those that describe color or physical deficiencies such as *aswad* ‘black’ and *aʔradj* ‘lame’ (see Hasan, 1986: 796 in Standard Arabic).

9 See Abu Helal (2021a) and Elghamry (2003) for evidence from Levantine and Standard Arabic, respectively.

10 See Abu Helal (2019) for an illustration of how Agree operates in the syntax of Arabic.

11 The reader is referred to Bhatt (2002), Hulsey and Sauerland (2006) for more discussion on adjectival modification in relative clauses.

12 The derivation in (6) proceeds along the following steps: first, the head NP [Op longest book] undergoes successive cyclic movement as in (i). (i) the [[long book] [[Op longest book] that John said [[Op longest book] that [Tolstoy wrote [Op longest book]]]]]. Second, the higher copies delete as in (ii): (ii) the λx [ that John said that [ Tolstoy wrote [Op longest book]]] Third, the low copy is interpreted via Trace Conversion (Fox 2002). Trace Conversion is a mechanism that converts movement copies into definite descriptions of type e using two derivational steps: first, variable insertion turns [Op longest book] into (Op) [ longest book λy(y = x)] where book and the inserted variable [λy(y = x], being predicates of the same type (i.e., type <et>), combines via Predicate Modification (Op) [λy [ longest book & (y = x)]. Second, a determiner replacement operation applies in such a way that the Op is replaced by a definite description operator to yield the definite description ιy [λy [longest book & (y = x)] of type e.

13 Bhatt (2002) assumes a 2-place predicate for the-est operator as represented in (Heim 1999): (i) ⟦ -est ⟧ = λG_<dt,t>_ λP_<dt>_. ∃d [ P(d) ∧ ∀Q ∈ G [ Q ≠ P→ ¬ (Q(D))] The operator in (i) takes two arguments: a contextually determined comparison class complement that denotes a predicate of predicates of degrees of type <dt,t> and a predicate of degrees that is lexically derived by the QRing of –est of type <dt>. When these two arguments saturate the –est operator, the superlative asserts that the relevant member has a length to a degree d that no other element in the comparison class has.

14 On the assumption has the superlative operator is lexically represented as the 3-place predicate (i). This operator takes three arguments: an individual-denoting object G that stands for the contextually determined comparison class complement, a relation of degrees and individuals P that is represented by a gradable adjective and an individual denoting x. When it applies to its arguments, the superlative delivers the truth conditions that there is a degree d such that the individual x has a property P to that degree and all the other individuals in the contextually determined comparison class G don't have the property to d (See Romero 2011 for an assessment of the superlative as a 2-place and 3-place predicate). i. ⟦ -est ⟧ = λG_<e,t>_ λP_<d<et>_ λx. ∃d [ P(d) (x) ∧ ∀y ∈ G [ y≠ x→ ¬ (P(d) (y))]

15 Heycock (2005) supported a non-reconstruction-based analysis: she argued that reconstruction falls short of predicting the much more restricted distribution of low readings of adjectival modifiers such as *first, last*, and *only*. According to Heycock (2005), the low reading in examples like (18) emerges in contexts where negative entailments license negative polarity items (NPI) and what Horn (1989) referred to as ‘short-circuited implicatures’ which capture the Neg-Raising phenomenon (NR). Interestingly, Heycock (2005) showed that the low reading of adjectival modifiers disappears in contexts that blocks NR such as factives, implicatives and epistimics, meaning that the low reading is derivable as the result of interpreting the negation in the entailment with lower copy (Heycock 2005: 263).

16 The analysis of Ouhalla (2004) and Aoun et al. (2010) is based on Kayne's (1994) theory of N-initial relatives. Ouhalla (2004), however, proposed a slightly different version of head-raising analysis: he assumed the raising structure of the *(ya)illi* relative along with the assumption that the relative clause is located in a left-branching Specifier position of N and hence it doesn't right-adjoin to NP. The N undergoes head movement into D to derive the Det-N-initial order.

17 Recently, the checking theory based on the Spec-head relation has been criticized and refined. The updated version of the theory incorporates notions such as valuation and interpretation which make use of a probe-goal relation with an EPP induced movement (Pesetsky and Torrego 2006). The updated theory also reduces the superfluous local spec-head domain to the c-command domain relation (Chomsky, 2005). For our purpose, the implementation of whatever minimalist version of Agree-Move theory leads to the same conclusion.

18 See Abu Helal (2021a) for a set of state-of-the-art diagnoses that confirm the fact that the *yalli* relative clause is unambiguously head external structure, including idiomatic expressions, definiteness, binding-theoretic facts, de re reading, and extraposition.

19 Hallman (2016: 1317–1326) discussed similar facts about the superlative morpheme aktar (sey) in Syrian Arabic. Hallman presented a set of diagnoses (e.g., islandhood conditions) confirming the fact that the dependency between the superlative operator and its scalar term is based on movement. He argued that the superlative operator aktar (ʃey) is base-generated in its surface position, indicating that the movement relation is derived by a null degree operator.

20 Unlike A-bar movement, movement deletion may involve less strict meaning identity for the internal representation of the relativized external NP than that which is encoded by the trace of A-bar movement in the reconstructed position (Sauerland, 2004).

21 Hallman (2016) documented and analyzed similar facts about the superlative aktar in Syrian Arabic, a form of Levantine Arabic. He observed that the superlative operator aktar (šey) never reconstructs below its surface position. At the same time, Hallman (2016) presented supporting evidence from pronominalization. islandhood conditions, Kennedy's Generalization in favor of a movement dependency between such a degree operator and a degree argument that is local to its scalar associate. These observations represent a puzzle: the absence of reconstruction of the degree operator is unexpected under the movement theory of the superlative. To resolve the puzzle, Hallman (2016) proposed that the degree operator is based generated in its surface position and the degree operator-variable dependency is derived via the movement of a null degree operator.

22 See Fox (1999, 2002) and Sportiche (2005, 2006) for an argument about the copy theory of movement.

23 Labeaux (1988) explains the asymmetry between adjuncts and complements as exemplified in (12) by proposing that adjuncts, but not complements, can merge late. Accordingly, complements must merge prior to movement as required under Projection Principle in which the selectional property of lexical items must be satisfied throughtout derivation (Chomsky, 1981). Adjuncts, on the other hand, do not need to satisfy subcategorization property and hence they may merge countercyclycally.

24 since it takes as its first argument a sentential element (Takahashi and Hulsey 2006).

25 On a minimalist approach to Case (Chomsky, 1998, 1999, 2001), structural Case assignment is achieved structurally by a Goal-Probe Agree operation based on c-command relation. Accordingly, the case-marked expression that enters the derivation with an unvalued Case feature serves as Goal for a c-commanding functional head which serves as a probe that values the Case feature of that expression by virtue of agreement (i.e., in phi-features such as person, number, and gender).

26 with the low reading.

27 We assume an N′-internal PRO movement within NP that leaves a trace t_2_ of type e. Such an empty category is to be focus-marked to shape the default absolute reading (Heim 1999; Krifka, 1998; Romero 2011).

28 The same semantics applies to derive the absolute low reading in the G-superlatives.

29 Notice that the *yalli* relative clause is argued to be nominal, not sentential so it needs Case.
